# How Does Social Media Influence People to Get Vaccinated? The Elaboration Likelihood Model of a Person’s Attitude and Intention to Get COVID-19 Vaccines

**DOI:** 10.3390/ijerph19042378

**Published:** 2022-02-18

**Authors:** Ammar Redza Ahmad Rizal, Shahrina Md Nordin, Wan Fatimah Wan Ahmad, Muhammad Jazlan Ahmad Khiri, Siti Haslina Hussin

**Affiliations:** 1 Centre for Research in Media and Communication (MENTION), Faculty of Science Social and Humanities, Universiti Kebangsaan Malaysia, Bangi 43600, Malaysia; 2 Centre of Social Innovation, Institute of Sustainable Building, Universiti Teknologi PETRONAS, Bandar Seri Iskandar 32610, Malaysia; shahrina_mnordin@utp.edu.my (S.M.N.); fatimhd@utp.edu.my (W.F.W.A.); 3 Faculty of Language and Communication, Universiti Malaysia Sarawak, Kota Samarahan 94300, Malaysia; akmjazlan@unimas.my (M.J.A.K.); hhaslina@unimas.my (S.H.H.)

**Keywords:** social media, COVID-19, vaccine, elaboration likelihood model, Twitter, factorial design, decision making

## Abstract

The global COVID-19 mass vaccination program has created a polemic amongst pro- and anti-vaccination groups on social media. However, the working mechanism on how the shared information might influence an individual decision to be vaccinated is still limited. This study embarks on adopting the elaboration likelihood model (ELM) framework. We examined the function of central route factors (information completeness and information accuracy) as well as peripheral route factors (experience sharing and social pressure) in influencing attitudes towards vaccination and the intention to obtain the vaccine. We use a factorial design to create eight different scenarios in the form of Twitter posts to test the interaction and emulate the situation on social media. In total, 528 respondents were involved in this study. Findings from this study indicated that both the central route and peripheral route significantly influence individually perceived informativeness and perceived persuasiveness. Consequently, these two factors significantly influence attitude towards vaccination and intention to obtain the vaccine. According to the findings, it is suggested that, apart from evidence-based communication, the government or any interested parties can utilize both experience sharing and social pressure elements to increase engagement related to COVID-19 vaccines on social media, such as Twitter.

## 1. Introduction

The global pandemic of novel coronavirus disease (COVID-19) has entered a new phase where vaccines against the disease have been developed, approved, and administered for the masses all over the world. It is reported, that until January 2022, 9.95 billion doses of vaccines have been administrated and 4.11 billion persons have been fully vaccinated worldwide [1]. Despite the huge numbers, in relative, there are only 52.7% of the world’s total who has completed the vaccination doses. It should be noted that several factors might lead to the small percentage of global vaccination rates, such as the limited number of supplies, logistics and procuring issues, and a country’s low purchasing ability contributing to the number of those vaccinated [2].

However, one of the important aspects that need to be highlighted when discussing the low vaccination rate is vaccine hesitancy. Multiple studies have reported vaccine hesitancy amongst the general public across different countries. For instance, it is reported that COVID-19 vaccines hesitancy is more than 40% in Italy [3]; more than 40% in both France and Poland [4]; and around 40% in Hong Kong [5]. The hesitancy to be vaccinated provide a great challenge to global stability as globalization demands full mobility of human being to nurture political, economic, and social stability throughout the world [6]. The presence of COVID-19 has caused global shock and disrupted some major economic sectors such as airlines and tourism [7]. Moreover, a recent study highlighted that slow vaccination rates might hinder the global “herd immunity project” against the COVID-19. Consequently, it might contribute to the existence of the newly identified COVID-19 variant [2] which might require another dosage of vaccine. If this situation continues, humanity will be trapped in a vicious cycle that is detrimental to civilization and world progress.

Henceforth, there is a need to overcome this vaccine hesitancy worldwide. It is reported that vaccine hesitancy is worsened by the existence of social media. Social media as a liberalized space produce both positive and negative outcomes. Twitter, for instance, as one of the major social media with 186 million users worldwide, is notoriously known as a platform where users spread unverified and false information [8,9]. This phenomenon is not unique to COVID-19. Previously, social media such as Twitter, provide a platform where rumors and misinformation on other epidemics, such as Zika, Ebola, and Yellow Fever, can be found [10]. Scholars across various disciplines have been promoting more research related to “hi-tech” misinformation to further understand how communication in Twitter affects its users’ behavior and ultimately how this can be used to overcome the problem of false information [11]. Moreover, medical professionals acknowledge that health communication via Twitter is becoming more important nowadays [12]. This emphasizes the need to produce a counter-narrative as an instrument to combat the situation of false information spread in micro-blogging platforms. Consequently, it can reduce vaccine hesitancy and ease the implementation of the global vaccination program.

However, there is still a limited number of studies conducted in understanding the effect of various communication approaches in conveying the information of COVID-19 vaccines towards its user attitude, intention, and behavior. Determining how to enhance vaccine acceptance through social media such as Twitter is important. Accordingly, questions relating to how people evaluate a tweet on the COVID-19 vaccine and how this evaluation affects their decision and behavior need to be examined. Normally, information sharing in the Twitter space is varied. Users might encounter tweets comprising of evidence-based information [13] where it contains detailed information about the COVID-19 vaccine. However, users might also encounter tweets that contain information related to the COVID-19 vaccine in tweets where other users share their experience when getting vaccinated. This experience-based information sharing is rich with storytelling approaches [14]. Moreover, users might also be affected by other elements which are socially grounded, such as whether a profile is verified, as well as the number of “Retweet” (RT) and “Likes” in a particular tweet [15].

To verify how these multiple factors can affect a person’s decision, our study conducted a factorial design to further understand how people react to different social media content related to COVID-19 vaccines and vaccination. We used the elaboration likelihood model (ELM) as the overarching theory in answering this research question [16]. The ELM model enables the explanation of various persuasive communication on a person’s decision and behavior. Based on both central processing and peripheral processing route in ELM, this study argues that evidence-based tweets will follow the central processing route while experience-based and social pressure follows the peripheral route. Both routes were argued to affect users’ decisions and behavior on COVID-19 vaccines.

The outline of this article is as follows. First, we discuss the current literature related to ELM and users’ behavior on COVID-19 vaccines. Based on that, hypotheses and conceptual frameworks for this study are developed. Then, we present our methodology in conducting this study followed by the presentation of findings. Lastly, the following section discusses the findings, limitations, and future recommendations.

This study is significant to academicians, medical professionals, and policy makers. First, it provides a ground of reference on how various persuasive communication, especially experience-based communication, can affect the user’s decision to obtain COVID-19 vaccines. Based on this, literature related to the effect of communication and vaccine acceptance can be expanded. Moreover, medical professionals and policy makers can use the findings reported in our study as a guideline to construct and develop communication strategies to further reduce vaccine hesitancy amongst the public. This will enable the global COVID-19 vaccination program to rapidly progress and will further improve the vaccination rate worldwide.

## 2. Literature Review

### 2.1. Disseminating Vaccine Information through Twitter

The need to increase communication intensity in spreading information about the COVID-19 vaccine is becoming more imperative in recent days. The method of communication has shifted from entirely relying on public and mass communication channels, such as newspapers, television, and radio, towards social media platforms, such as Facebook, Twitter, and YouTube [17]. Contrary to traditional mass media where communication originated from a well-structured news agency, social media comprises affordances that allow the public and individual users to become the source of information [18]. Furthermore, the technological affordances and social networking nature of social media enable other users to share the information throughout their network of connections [19]. In traditional media, any new information will be filtered by various editors before it is conveyed to the public. In contrast, users of social media freely share any information without obligation to verify the information [20].

This phenomenon, together with the rapid increment of internet users, has turned social media into a “double-edged sword” where it can strike both essential and fake information towards the masses. In the context of vaccines, scholars argue that there is a need to develop a new messaging strategy to overcome false information and vaccine hesitancy on social media [21]. The two most highly consumed platforms in spreading and accessing information on social media are Facebook and Twitter [17,22]. Despite Facebook having more users compared to Twitter, scholars found out that Twitter is a more vulnerable platform that easily exposes the threat of false information and spread of hesitancy. In a study comparing both social media platforms, Yang et al. (2021) identified Twitter as having a lower credit score compared to Facebook [22]. Moreover, Twitter is also identified as a sentinel tool to monitor public opinion of COVID-19 vaccination [23]. The combination of the Twitter algorithm and its micro-blogging affordances has enabled Twitter to become a social media platform that can spread information rapidly [24]. Therefore, there is a need to conduct this study based on the context of Twitter as a platform for disseminating information about the COVID-19 vaccine.

### 2.2. Elaboration Likelihood Model (ELM)

The process of decision making for any health decision including vaccination is a decision made under risk consideration where it involves a choice between prospects and gambles [25]. Debates on determining theoretical perspectives to explain the decision-making process in health communication has continuously occurred. Amongst the frequently utilized theory are the expected utility theory and the prospect theory [25,26]. Besides that, scholars also used other behavioral theories based on a person-perceived benefit or value when making a health-related decision. It included the theory of reasoned action [27] and the theory of planned behavior [28]. However, the mechanism of these theories is mostly based on message framing and behavioral consequences. For instance, prospect theory involves a person valuing its given option such as “What option do I have in overcoming COVID-19”. The person will then make the decision based on the highest value option [26]. The drawback of this theoretical perspective is that we cannot understand how persuasive communication, which can be abundantly found on social media, influences a person’s decision making and how the process can be explained.

In the field of communication study, the elaboration likelihood model (ELM) has been used to explain the process of communication in influencing a person behavior and decision making. The basic premise of ELM is to understand how persuasive communication can influence a personal attitude and behavior where the process is explained by how likely the person will elaborate upon (i.e., think about) the given communication [16,25]. It is through this theoretical perspective that we will be able to understand the effect of different types of persuasive communication on the person’s attitude and behavior regarding COVID-19 vaccines.

The ELM model argues that the elaboration process occurs in a continuum within a person cognitive function in a “communication-induced attitude change” [16]. The continuum can be divided into dual routes known as the central and peripheral routes. The process of a person elaborating the persuasive communication is based on their cognitive information processing and degree of elaboration [29]. When a person has carefully and thoughtfully considered the given communication based on merit, it is considered to have taken the central route. The person is considered to elaborate based on the peripheral route when a decision is made based on a simpler cue (e.g., attractive and colorful message), rather than scrutinizing the true merits of the presented communication [16,30].

Various studies across multiple disciplines have used the ELM model to explain a person’s decision making. A plethora of studies have been conducted in the marketing realm where scholars look into the influence of different types of marketing communication strategies on consumer purchasing decisions [29,31]. It is also being used in political communication studies to understand how a person reacts to different political speeches, media campaigns, and manifestos of political parties [32,33]. In the realm of health communication, ELM has been used in various fields, such as understanding the effectiveness of tobacco package warning labels [34], health promotion by using radio dramas [35], and public health communication for safer sex practices [36]. Despite the various empirical studies, there is still limited study in the context of COVID-19 vaccination and social media. As discussed earlier, this phenomenon is both emerging and important as this study will answer the question of how to develop a communication strategy that can increase public awareness and trust in the health systems. We use ELM as the underpinning model for this study. In doing that, we have to first discuss on what is the common persuasive communication found in Twitter and how we can categorize and fit it into the dual processing model of ELM. The following section provides this discussion.

### 2.3. Central Route Factors

Quality of arguments is an essential element in persuasive communication where it triggers relatively primitive affective states that become associated with attitude objects [16]. Having a high-quality argument represents high information reliability and persuasive strength of an argument which are essential elements for the central route [29]. Persuasive strength in the context of communication within social media and Twitter refers to information provided in the tweet [37]. Some scholars perceived that in measuring persuasive communication in the context of the information provided, attention needs to be given to the ability to comprehend information [38]. However, the ability of a person to comprehend information depends on the quality of the information which can be described into two elements: information completeness and information accuracy [29,39,40]. This study adopted both elements as central route factors.

Information completeness is referred to when there is sufficient depth and breadth of information in communication [29]. In this case, Twitter is used as the social media platform that applies the concept of communication. Information completeness can be understood as the degree where information about the vaccine is provided to its actual status, as perceived by the public [13]. As the objective of the COVID-19 vaccination is to overcome COVID-19 disease itself, the purpose of vaccination and the mechanism of vaccination are essential pieces of information. An AA study conducted on information elaboration about childhood vaccination in online parenting by Goh and Chi [41] reported that parents primarily seek to understand the purpose of vaccination on their children (Goh & Chi, 2017). On the other hand, in relation to vaccination information vaccination, another scholar argues that there is a need to include information related to the effectiveness of vaccines [13]. Henceforth, in this study, we argue that both the purpose and effectiveness of information are crucial when it comes to information completeness. However, in the context of effectiveness, as COVID-19 vaccination is still currently administered during the execution of this study, providing effectiveness based on the effectiveness of previous vaccines in combating viral diseases such as Polio or Measles would be necessary. In addition, the past study also indicates that the presence of infographics would be beneficial in conveying information [29].

Besides information completeness, the other important element is information accuracy. An empirical study has shown that individual decision making depends on obtaining correct and accurate information as it allows them to separate between true and fake messages [40]. Information accuracy for this study is defined as the degree to which information is correct, accurate, and unambiguous [29,42]. Accurate information needs to show interrelation and consistency between provided data and reality [43]. In the context of vaccine communication, information accuracy requires all the discussed information to be accurate and consistent. Scholars argue that to increase the effectiveness and accuracy of information, especially when it is related to social media and vaccines, attention needs to be given to the source of communication. Information coming from an authoritative source, such as a department or ministry of health, will be more accurate [21,44]. Therefore, the information is proven to be correct, credible, and believable, and this study used the information provided by the Malaysia Ministry of Health to ensure the information accuracy is preserved.

### 2.4. Peripheral Route Factors

According to the elaboration of likelihood model, besides argument quality representing the central route in influencing attitude, the peripheral route is best explained by other “cues” that affect the individual attitude through bypassing the argument processing [16]. Scholars argue that the cue is operative when the individual is being “unmotivated by the subjects or is unable to process the issue-relevant arguments”, abundantly found in the central route [16]. Therefore, the individual looks for simpler cues, such as source credibility, aesthetics, and popularity [29]. 

Experience Sharing is one of the emerging communication approaches to overcome barriers and limitations in the talk between patient and physician is experience-based communication. Experience-based communication in the context of health communication refers to the role of experience as a central core in communication information based on storytelling techniques [45]. Scholars have reported that communication via storytelling is becoming more important, especially in the era of social media [46]. The power of storytelling as a health promotion tool is based on the ability of the storyteller to create an emotional connection that not only enables information sharing but also creates emotional benefit and therapeutic effects [47]. This is in line with the peripheral route, as several scholars argue that the route mostly depends on an individual’s emotion rather than argument quality to influence their decision-making process [16,37,48]. Therefore, in the context of this study, a Twitter post shall contain information related to the COVID-19 vaccines based on storytelling and experience sharing. It will lead readers to perceive the post has a high experience sharing.

Social Pressure—Petty and Cacioppo have indicated that the social factor is one of the important elements in a peripheral cue that influence and affect individual decision-making [16]. Furthermore, scholars who studied social phenomena during the COVID-19 pandemic have suggested that social factors are an essential construct that might interact with other constructs as a mediator or moderator [49,50,51]. For instance, Jiang et al. (2021) reported that social norms negatively moderate the mediation relationship between news attention and social distancing behavior, indicating that social factors influence individual behavior despite the risk of getting the disease. This finding is further enhanced by several studies that reported that social pressure can be used as a medium in creating individual compliance towards COVID-19 prevention strategies by the authority [52,53]. Accordingly, social pressure can be included as a message in social media posts, such as Twitter. It needs to be framed to promote vaccination based on relationship value and social pressure [54]. Furthermore, credibility has been known to be an essential element in social media. Several studies have identified that Twitter posts that contain credible elements, such as a verified profile, as well as a high number of retweets and likes, tends to get more attention and could create a sense of awareness to its readers [10,55]. Eventually, this creates social pressure and might influence individual decision making in the context of vaccination. Therefore, this study viewed Twitter posts with high social pressure to contain both relationship value and credibility.

### 2.5. Perceived Informativeness

Previously, we have discussed the literature associated with ELM and how persuasive communication in the context of social media posts (i.e., Twitter) can be categorized into a central route factor and a peripheral route factor. However, it is essential to identify the working mechanism on which both persuasive communications will be able to affect and influence the cognitive ability of the individual. Consequently, the mechanism will be able to explain the communication influence on attitude and behavioral intention, which is monumental in further understanding behavioral change in getting vaccinated during a pandemic.

Perceived informativeness refers to a condition relating to an informational concept where an individual can equip the information required with the information provided [56]. Accordingly, readers who read the Twitter post will consider it suitable if the information they required about the vaccines is stated and provided in the post. This concept is also echoed by other scholars in the vaccination study. Rzymski et al. (2021) show that information is an essential element in developing communication strategy and getting the public well informed about the vaccination. Furthermore, a similar situation is also reported in other realms involving social media. Chang et al. (2020) reported that a social media post is needed to be measured through its capability to provide ample information to the users.

As the central route factor of the ELM in the context of this study is categorized into information completeness and information accuracy, both can provide the required information needed by the readers. Furthermore, the scholar also indicated that there is a need to emphasize the accuracy of information even though the communication is made for the public. It includes stating information related to vaccines and the disease it is intended to control [57]. Therefore, when both accuracy and information are high in a Twitter post, readers will perceive that they are well informed in regards to the COVID-19 vaccine. On the other hand, if accuracy and completeness are low, the readers will believe that they are inadequately informed. Based on the argument, the first hypothesis for this study is provided below.

**Hypothesis 1 (H1).**  *Central route factors, which are information completeness and information accuracy, have a positive relation to perceived informativeness where high information completeness and information accuracy have stronger effects on perceived informativeness than low information completeness and information accuracy in the context of Twitter posts and COVID-19 vaccines.*

Besides central route factors, perceived informativeness could be also influenced by peripheral route factors. The peripheral route in the context of this study refers to a form of communication that contains high experience sharing and high social pressure. Scholars have indicated that the individual would be able to obtain information through the process of narration and storytelling. They argue that people, especially laypeople, are unable to appreciate and understand information conveyed in the form of statistics and figures. Instead, information especially related to health communication can be disseminated in the form of narration and storytelling, such as in testimonials [47,58]. Experience shared in the Twitter post is deciphered by the readers as its storytelling nature can enable readers to empathize and project it as if they are the one who experiences it. Furthermore, a peripheral route factor indicates that social pressure is an important element. This enhances their ability to obtain information in the Twitter post which leads to perceived informativeness, as discussed above. Therefore, the second hypothesis of this study is provided below.

**Hypothesis 2 (H2).**  *Peripheral route factors, i.e., experience sharing and social pressure, have a positive relation to perceived informativeness where high experience sharing and social pressure have stronger effects on perceived informativeness than low experience sharing and social pressure in the context of Twitter posts and COVID-19 vaccines.*

### 2.6. Perceived Persuasiveness

The other element that is important in influencing attitude and behavioral intention is perceived persuasiveness. In the context of communication study, it refers to individual beliefs or behavior that is changed rationally and sensibly [29,59]. Several theoretical perspectives in the communication context highlighted that the ability of messages in communication to unlock the cognitive function in the individual might explain the attitudinal change [28,60]. The cognitive function refers to the ability of readers to observe and decipher the given stimuli [61]. Besides, perceived persuasiveness can only occur when the readers have a sense of trust and belief in the information conveyed to them [62,63]. Hence, in this context, it is argued that central route factors will be able to influence the readers perceived persuasiveness. Both information completeness and information accuracy are crucial in creating a sense of trustworthiness. Information accuracy, for instance, is essential in keeping the communicated information believable and correct. Thus, a highly accurate Twitter post will cause high strength of persuasiveness amongst the readers. In contrast, if the information is inaccurate and fake, it will not be believable and there will be no sense of trustworthiness. Thus, the third hypothesis for this study is provided below.

**Hypothesis 3 (H3).**  *Central route factors, i.e., information completeness and information accuracy, have a positive relation to perceived persuasiveness; in other words, high information completeness and information accuracy have stronger effects on perceived persuasiveness than low information completeness and information accuracy in the context of Twitter posts and COVID-19 vaccines.*

Perceived persuasiveness is also argued to be positively influenced by the peripheral route factors. In several studies involving Twitter as the context, it is reported that certain factors, such as verified profiles and the number of likes and retweets, create a sense of trustworthiness amongst the readers [64]. Moreover, the combination between experience sharing based on storytelling and social pressure might create a synergistic effect in convincing and influencing the readers as well as attracting their belief [59,65]. Furthermore, a Twitter post with a normal profile and less social pressure in the post is going to be viewed as a normal post and it is unable to project a sense of belief amongst the readers. Therefore, it is hypothesized as shown in the fourth hypothesis below.

**Hypothesis 4 (H4).**  *Peripheral route factors, i.e., experience sharing and social pressure, have a positive relation to perceived persuasiveness whereas high experience sharing and social pressure have stronger effects on perceived persuasiveness than low experience sharing and social pressure in the context of Twitter posts and COVID-19 vaccines.*

### 2.7. Attitude and Behavioural Intention towards Vaccine

The concept of attitude, as discussed in the realm of social psychology, refers to the individual attitude which generally posits a negative or positive evaluation of acting a behavior [66]. Connecting with the behavior, Moore and Lucas [67] mentioned an essential measure for the actual behavior is the attitude an individual has concerning an action to be executed (e.g., by considering taking the vaccine). Empirical evidence shows that communication elements, including both information and persuasiveness, can influence attitude change, regardless of stronger or weaker affective cues (i.e., central or peripheral route) [16]. Thus, in the context of this study, both route factors influence individually perceived informativeness and persuasiveness relating to COVID-19 vaccines. These two constructs affect the individual attitude towards the vaccines. Based on the arguments, the fifth and sixth hypotheses for this study are provided below.

**Hypothesis 5 (H5).**  *Perceived informativeness will affect individual attitudes towards the COVID-19 vaccine.*

**Hypothesis 6 (H6).**  *Perceived persuasiveness will affect individual attitudes towards the COVID-19 vaccine.*

Likewise, behavioral intention refers to the individual’s intention to get vaccinated. A study reported that the attitude of an individual will affect the individual’s intention in getting vaccination [68]. This is further explained in several theoretical studies where it is argued that attitude acts as a predictor towards intention by emphasizing the positive relation of the behavior and how it will benefit the individual. Furthermore, the attitude creates an evaluation and expectation process which is translated into the individual’s intention [66]. Therefore, the seventh hypothesis for this study can be found below.

**Hypothesis 7 (H7).**  *Attitude towards vaccines will affect individual intention to get the COVID-19 vaccine.*

**Figure 1:**
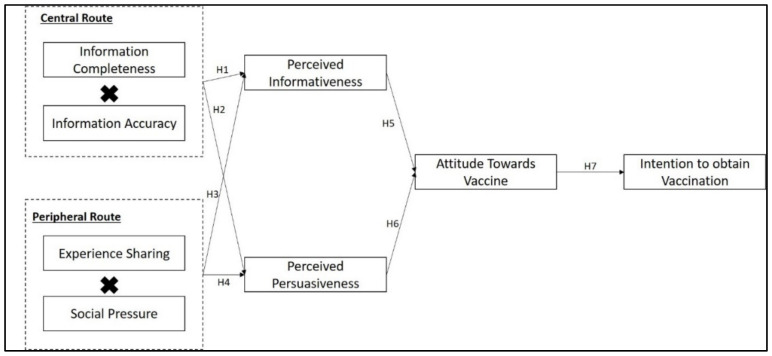
Conceptual framework of the study.

Based on the discussed literature and developed hypotheses, the conceptual framework for this study is shown in Figure 1 below.

## 3. Materials and Methods

This study employed four different 2 × 2 factorial designs. The factorial design allowed us to investigate any interaction effect between information completeness and information accuracy. Similarly, the design also enabled an investigation of the interaction effect between experience sharing and social pressure. Consequently, we could identify both central route and peripheral route effects on perceived informativeness and perceived persuasiveness. Definition and description of the constructs are included in the Supplementary Materials.

Respondents in this study were instructed to view a screenshot of a Twitter post based on a different scenario which will be discussed in detail in the next section. This defines the type of communication regarding COVID-19 vaccination that they might encounter on social media, especially on Twitter. Respondents were asked to answer a series of questionnaires which were used as the instrument for this study. The questionnaire was based on a 5-point Likert scale and it was adopted and adapted from previous studies [17,29,69,70,71,72,73,74]. Details of the instrument and sources can be found in the Supplementary Materials.

### 3.1. Scenario Design

The design of scenarios in this study was based on studies using ELM and 2 × 2 factorial design where the scenarios are comprised of two different levels (high and low) for each factor, according to the elaboration route (i.e., central, or peripheral) [29]. The central routes were divided into four different scenarios: high information completeness and high information accuracy, high information completeness and low information accuracy, low information completeness and high information accuracy, and low information completeness and low information accuracy. On the other hand, the peripheral route also had four different scenarios: high experience sharing and high social pressure, high experience sharing and low social pressure, low experience sharing and high social pressure, and low experience sharing and low social pressure. Details of the scenario are also shown in the Supplementary Materials.

**Figure 2:**
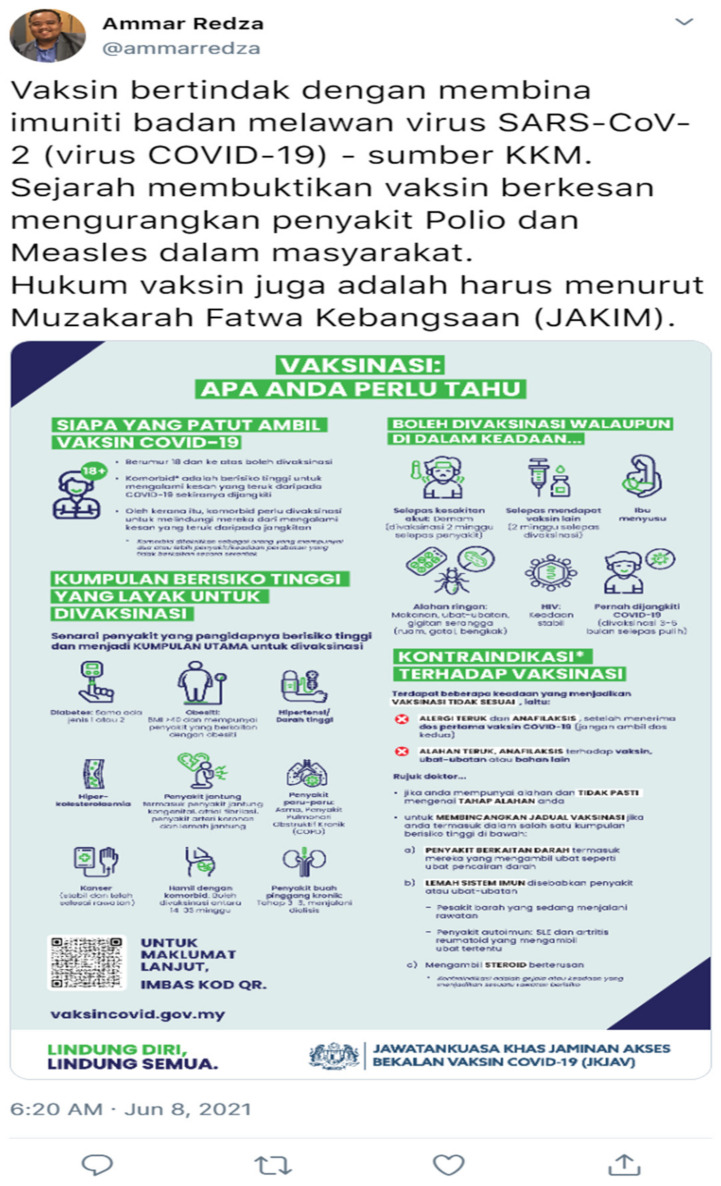
Scenario 1 (high information completeness and high information accuracy).

**Figure 3:**
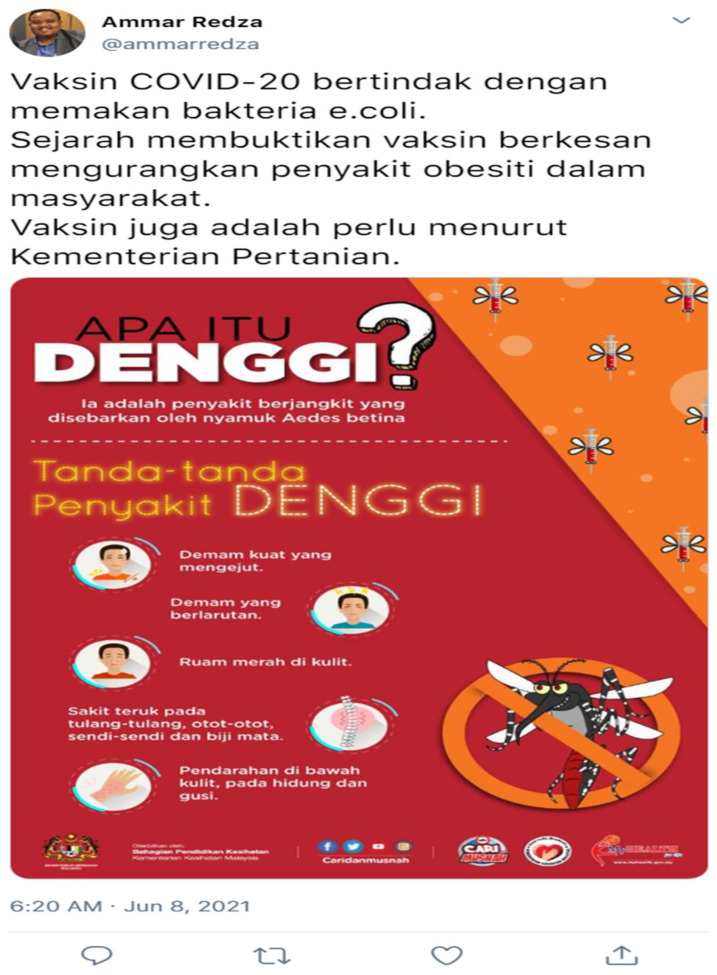
Scenario 2 (high information completeness and low information accuracy).

The scenario was designed to represent a single Twitter post. In total, there were eight different Twitter posts. The official language of Malaysia, i.e., “*Bahasa Melayu*”, was used to construct the post. The post was not be distributed to the public but, instead, screenshots are taken and used as the stimulant for this study. To maintain consistency, a similar user was used for each Twitter post reflecting each scenario used in this study. As for information completeness and information accuracy, the information was obtained from publicly accessed and distributed information about COVID-19 vaccines by the Malaysia Ministry of Health. The example of the Twitter post screenshot depicting scenarios 1 and 2 is shown in Figure 2 and Figure 3, respectively.

The English translation for the text inside Figure 2 is as follows: “Vaccine works through developing our immunity to fight virus SARS-CoV-2 (COVID-19 Virus)—source MOH (Ministry of Health). History has shown how the vaccine can reduce Polio and Measles spread in society. The National Fatwa Committee ruling for vaccination is Harus (recommended)”. While the English translation for the text inside Figure 3 is as follows: “Vaccine COVID-20 act by eating the e-Coli bacteria. History has shown how the vaccine is effective in reducing obesity amongst society. According to the ministry of agriculture, the vaccine is a must”. The other scenarios and their respective English description for each scenario are included in the Supplementary Materials.

A high information completeness scenario contained information on vaccine mechanism, past evidence of effectiveness and an infographic poster from the Malaysia Ministry of Health (MOH). High information accuracy means that all the information provided is accurate and correct, based on information by the MOH. Low information completeness means that some of the information is omitted while low accuracy is manipulated by inputting information with significant error (e.g., mentioning COVID-20 instead of COVID-19 and mentioning bacteria instead of viruses). Meanwhile, high experience sharing contained experience-based communication and storytelling method while high social pressure is shown through a verified profile or a high number of retweets and likes. Furthermore, it also contains a prosocial responsibility. In contrast, low experience sharing contains no storytelling technique and no sharing of experience. Low social pressure is reflected based on no verified profile or a low number of retweets and likes, as well as no prosocial responsibility communication in the tweet. 

All the eight scenarios were presented to the respondent randomly where they could select which internet link they prefer. Here, the scenario was only revealed to them once they selected it. All the scenarios and instruments were tested for their reliability and validity through the pilot study, which will be discussed next.

### 3.2. Pilot Study

A pilot study was conducted in April 2021 before full-scale data collection to test both the reliability and validity of the instrument used and whether the scenario possessed the manipulation intended for it. In total, 160 respondents were involved in the pilot study. This study was conducted using an online questionnaire. Each scenario was placed in its respective link. The respondents were first brought to the landing page where they could freely select a randomly numbered link. The link brought them to the scenario and the respective questionnaire. First, they were introduced to the landing page where they could freely select the randomly assigned link. They were presented with the scenario and answered the following questionnaire. Apart from the 22 items, the instrument was equipped with 4 questions to test whether the respondent read the scenario carefully and whether the scenario was representative of the constructs measured.

Data collected from the study were then analyzed using reliability analysis—the Cronbach alpha score and the independent *t*-test for manipulation check. Findings indicated that there was significant difference between high and low manipulation groups—information completeness (F = 2.623, *t* = 3.458, *p* = 0.002); information accuracy (F = 6.230, *t* = 2.43, *p* = 0.021); experience sharing (F = 5.006, *t* = 2.244, *p* = 0.003); and social pressure (F = 6.135, *t* = 2.361, *p* = 0.028). Furthermore, all the constructs used in this study show high internal consistency (a Cronbach alpha score of more than 0.8), indicating that the instrument was reliable. The pilot study showed that the instrument used in this study could be used for the full-scale study.

### 3.3. Full Study

The full study was conducted within June 2021 comprising a total of 528 respondents. The sampling size was determined using G-power analysis to ensure the amount is sufficient for effect size and statistical power [75]. The study employed a randomized factorial design. First, respondents were recruited through voluntary participation where they clicked the link to the study via a post published on social media platforms. Respondents were then required to select a link containing a randomly assigned scenario. Before participating in the study, the respondents were presented with the consent form. They were informed of their right to not participate in this study and any data collected from them was under the legal framework under Malaysia Personal Data Protection Act (PDPA). Data collected were then analyzed for manipulation check, descriptive analysis, analysis of variance (ANOVA), and path analysis. The findings are presented in the next section of this article. 

## 4. Results

The demographical analysis shown in the Table 1 indicated that 45.7% of the respondent were male and 54.3% were female. The majority of the respondents were in the age between 30 and 49 years old (63.9%) and 84.7% of them attended secondary school education or higher. Furthermore, 45.4% of the respondents indicated that they do not have any preference regarding vaccine type/brand and 32.3% preferred the COVID-19 vaccine from Pfizer-BioNTech. There were also 16.8% of the respondents who mentioned that they were hesitant with the vaccine. In terms of time spent on social media, 38.6% stated that they spent an average of 1–3 h daily on social media and 46% of them spent more than 3 h daily on social media.

Table 2 shows the descriptive analysis of this study. Apart from that, we also conducted a manipulation check to ensure that the manipulation was well designed in the questionnaire. All the results of the manipulation were significant, indicating that there is a manipulation of the high and low level stimulants in the questionnaire. Information completeness (F = 16.294, *t* = 6.989, *p* ≤ 0.001); information accuracy (F = 24.485, *t* = 9.089, *p* ≤ 0.001); experience sharing (F = 32.558, *t* = 5.081, *p* ≤ 0.001); and social pressure (F = 26.111, *t* = 3.213, *p* ≤ 0.001).

### 4.1. ANOVA Test

To test the first four hypotheses in this study, we conducted a one-way analysis of variance (ANOVA) and Scheffe’s post-hoc tests using Statistical Package for Social Science (SPSS) version 21. The results from this are shown in Table 3. The findings showed that the central route factors were found to have a significant influence on perceived informativeness (F = 55.559, *p* < 0.001). Moreover, the mean score of the first scenario (high completeness × high accuracy, HH) was 4.294, which was the highest amongst the other condition for the central route, while the mean score of the fourth scenario (LL) was 2.377, which was the lowest of the four conditions for the central route. The result of Scheffe’s post-hoc test also showed a significant difference between each condition except between the second (HL) and fourth scenario (LL). It indicated that the first hypothesis (H1) is supported. Similarly, the second hypothesis (H2) of the result of Scheffe’s post-hoc test also indicated that there was a significant difference between groups and, therefore, that the second hypothesis (H2) is also supported.

Findings from the peripheral route factors were also found to have a significant influence on perceived informativeness (F = 14.836, *p* < 0.001) and perceived persuasiveness (F = 16.837, *p* < 0.001). In Scheffe’s post-hoc test, all the scenarios showed that there was a significant difference between each group. Hence, it indicated that the third (H3) and fourth (H4) hypotheses were supported. In viewing the mean score, the scenario with high experience sharing and high social pressure (S5) had the highest mean score for both perceived informativeness (M = 3.756) and perceived persuasiveness (M = 4.026). It showed that both high experience sharing and high social pressure had a stronger influence than the scenario that lacked both elements (S8).

### 4.2. Path Analysis

**Figure 4:**
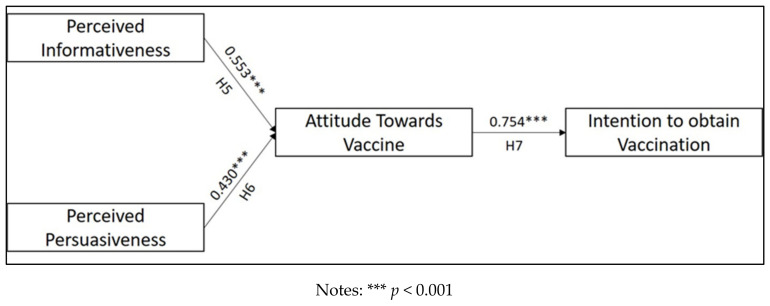
Path analysis of the study.

The path analysis using partial least square–structural equation modelling (PLS-SEM) was conducted to test the fifth, sixth, and seventh hypotheses, respectively. Smart PLS 3.0 was used to conduct the analysis. The factor loadings (CFA) for each measurement indicator for this study are shown in Table 2. Findings from the model fit test are shown in Table 4 which stated that the SRMR score for this model is 0.022. According to Hair et al. [76], the SRMR score of less than 0.08 indicated that the path model is fit. Furthermore, the path analysis shows that the standard coefficient score for the relationship between perceived informativeness and attitude towards vaccine (H5) was 0.553, with the t-value of 6.329 indicating that there was significant influence. Perceived persuasiveness was also reported to have a significant influence on attitude towards vaccine (r^2^ = 0.430, *t* = 4.969). The seventh hypothesis was also supported, showing that attitude had a strong influence on the intention to get vaccinated (r^2^ = 0.754, *t* = 30.894). The details of these findings are shown in Table 4 and Figure 4.

## 5. Discussion

### 5.1. The Effect of Central Route vs. Perceived Informativeness (H1) and Perceived Persuasiveness (H2)

The findings indicated that both H1 and H2 for this study were supported. The findings from this study were consistent in several other studies that employed ELM and tested the direct and indirect effect of the central route, namely information accuracy and information completeness on perceived informativeness and perceived persuasiveness [29,77]. Despite the fact that factorial effect of information accuracy and information completeness have been examined in past studies, most studies conducted are in the realm of marketing and consumer research. There is still no such study in our understanding that is conducted in the context of vaccination and public health management. The main findings in this study were that information accuracy is more crucial compared to information completeness. It indicated that information accuracy in tweets or any social media post is essential in building up public informativeness and persuasiveness. In contrast, complete information but without accuracy tends to reduce both factors. Cornwall (2020) reported that the public believes information coming from an authoritative agency to be accurate and trustworthy. These findings also showed that in the current situation of COVID-19, the public do not value false information, especially when the information originates from unreliable resources. This study, along with several other studies, reported that authentic information can educate the public on matters related to COVID-19, including vaccination [51,78].

### 5.2. The Effect of Peripheral Route vs. Perceived Informativeness (H3) and Perceived Persuasiveness (H4)

Both H3 and H4 were supported in this study. Firstly, the findings indicated that both experience sharing and social pressure, as well as its interaction, are expressed in the form of communication which influences both the perceived informativeness and perceived persuasiveness of the respondent. Consequently, the findings verified the mechanism of both informativeness and persuasiveness of a respondent in the context of the COVID-19 vaccine is explained through both central and peripheral routes in which the mechanism is based on ELM [16]. Moreover, the finding was in line with several past studies that showed how an individual was able to grasp critical information including related to healthcare through storytelling and experience sharing [45,46]. According to Briant et al. (2016), experience sharing focuses on storytelling where the emotional connection enabled people to appreciate other stories and garner the information from the story. Furthermore, the experience shared from a person to the other person through storytelling influences the person decision through its natural omnipresence property [47].

Besides experience sharing, the other essential component in the peripheral route is social pressure. This study echoed and re-emphasized how social pressure is an important element in public health management. For instance, personal attention towards the news and their social distancing behavior is reportedly mediated by social norms [51]. In addition, the mechanism of social pressure can influence both public informativeness and persuasiveness in the context of the COVID-19 vaccine and can be further explained by the current phenomenon of public health measures in fighting COVID-19. Certain specific measures, such as social distancing and mask-wearing, have created a new norm in which not adhering to it is a sign of abnormality in the eyes of society. This is in line with other studies that highlighted that social compliance could influence a person’s actions [52].

### 5.3. Perceived Informativeness and Perceived Persuasiveness towards COVID-19 Vaccine Attitude (H5 and H6)

Both H5 and H6 of this study are also supported. Similarly, another study reported how information and persuasiveness play an important role in determining an individual attitude towards the COVID-19 vaccine. For example, Piltch-Loeb et al. (2021) reported that the authenticity of information obtained through traditional and social media is one of the reasons that leads encourage COVID-19 vaccine acceptance. Moreover, persuasiveness has been shown to affect individual attitudes in this study. Perceived persuasiveness is the situation where an individual belief or behavior is changed rationally and sensibly [29,59]. Previous communication studies have shown how persuasion can change the human attitude towards anything [79]. In a way, findings from this study are aligned with other studies within the context of health communication. For instance, a study conducted in the United States, Palm, Bolsen, and Kingsland [54] shows that individual attitudes and acceptance towards vaccination are affected by how the media frames the message on vaccines towards them.

### 5.4. The Effect of Attitude towards COVID-19 Vaccine Behavioural Intention (H7)

The final hypothesis of this study is supported where the study has shown that individual attitudes affect their COVID-19 vaccine behavioral intention. The findings echoed several other studies in the context of the COVID-19 phenomenon where it is reported that attitude is the main contributor towards vaccine acceptance [5,80,81]. In addition, this study has identified that individual attitude is affected by media consumption. The content found inside the media can pursue users’ attitudes, either through the central route or the peripheral route. This further emphasizes the crucial role played by media, including social media, in determining individual interest to obtain COVID-19 vaccination. Moreover, findings from this study support the idea that pursuing individuals to get vaccination is not only dependent on scientific facts. Compelling storytelling and prosocial message are also capable of affecting the individual attitude towards COVID-19 vaccines. This shall expand the boundary for content creation by the media about promoting COVID-19 vaccines.

## 6. Conclusions

What motivated us in conducting this study is the current phenomenon of the mass vaccination process to overcome the COVID-19 pandemic and the presence of hesitancy amongst the members of the public in getting vaccinated. The existence of social media, such as Twitter, has made the situation worse where fake news and false information have spread rapidly [8,9]. Henceforth, this study was conducted to examine the factorial effect of both central and peripheral routes in the context of COVID-19 vaccines. We argue that it is critical to understand how a person interprets messages related to COVID-19 vaccines and how we can explain the ability to influence the behavior of a community in relation to the COVID-19 vaccination. 

Although numerous studies have adopted ELM as the main theoretical model, most of the studies conducted were in consumer and marketing studies [29,39,48]. In the health realm, most studies based on ELM focus on health literacy and health-related advertising [34,82]. Therefore, our study contributed to the expansion of literature particularly in health communication based on two aspects. First, it examined the factorial effect of information completeness, information accuracy, as well as experience sharing and social pressure on individual attitude when they were exposed to the associated stimuli. This reduced the current gap in health communication in the context of COVID-19 vaccination in understanding how various information triggers various mechanisms (i.e., perceived informativeness and perceived persuasiveness) in the individual. Secondly, this study provides further evidence on how this mechanism affects the individual’s attitude and behavioral intention towards the COVID-19 vaccine.

Consequently, the findings provided important information for practical application. In combating hesitancy on COVID-19 vaccines, which is reportedly a global problem [82,83], this study shows how the peripheral route has the same impact as the central route in influencing individual attitudes on the vaccine. Furthermore, our study shows that information in the form of experience sharing, containing social pressure indicators, significantly contribute to individually perceived informativeness and persuasiveness. Eventually, this can be adopted by policymakers and other related agencies in creating a variation in communication strategies to increase public acceptance surrounding the COVID-19 vaccine.

However, we believe that there are some limitations in our study which can be enhanced by future research. First, we do not consider the effect of hesitancy as a factor that might increase or decrease individual intention to be vaccinated. Therefore, future studies could investigate the moderating effect of hesitancy on this relationship. Second, our study focused on a textual stimulant as the medium for investigating the factorial effect. The usage of graphic-based and video-based social media has been increasing. This means that different types of stimulants can create different impacts. Thus, future studies should investigate the impact of various kinds of stimulants on the individually perceived informativeness and persuasiveness. Third, this study is conducted in the context of the Malaysian population. Our study did not investigate the influencing factor of other populations. Henceforth, a similar study should be conducted in other regions with different populations to assess the impact of culture on the factorial effect of the tested element in this study.

## Figures and Tables

**Table 1:** Demographic statistics of the respondents (*n* = 528).

Category		Percentage (%)
**Gender**	Male	45.7
	Female	54.3
**Age**	20–29	21.2
	30–39	31.4
	40–49	32.5
	50 and above	14.9
**Highest Academic Qualification**	Primary education and below	5.3
	High/secondary school education	42.5
	College diploma and above	42.2
**Preferred Vaccines**	Pfizer	32.3
	Astra Zeneca	10.5
	Sinovac	3.8
	No preference (any brand and type are accepted)	45.4
**Time spent on social media (Daily)**	Less than 1 h	15.4
	1–3 h	38.6
	3–5 h	31.2
	More than 5 h	14.8
**Vaccine hesitancy**	Yes	16.8
	No	83.2

**Table 2:** Descriptive analysis for each construct and CFA.

Description/Item	Mean Score	Standard Deviation	Factor Loading (CFA)
**Perceived informativeness (PInf)**			
**PInf1**	3.636	1.3759	0.968
**PInf2**	3.617	1.3857	0.955
**PInf3**	3.535	1.4167	0.961
**PInf4**	3.208	1.4395	0.959
**PInf5**	3.112	1.4807	0.948
**PInf6**	3.320	1.4293	0.970
**Perceived persuasiveness (PPer)**			
**PPer1**	3.524	1.3797	0.972
**PPer2**	3.435	1.3593	0.843
**PPer3**	3.498	1.3443	0.973
**PPer4**	3.513	1.3663	0.972
**PPer5**	3.524	1.3302	0.959
**Attitude towards COVID-19 vaccines (Attitude)**			
**Attitude 1**	3.572	1.3312	0.974
**Attitude 2**	3.714	1.3577	0.954
**Attitude 3**	3.468	1.3822	0.968
**Attitude 4**	3.543	1.3547	0.960
**Attitude 5**	3.625	1.3524	0.971
**Attitude 6**	3.617	1.3224	0.978
**Attitude 7**	3.480	1.3662	0.977
**Intention to vaccinate (ItV)**			
**ItV1**	3.621	1.3389	0.976
**ItV2**	3.643	1.3533	0.983
**ItV3**	3.606	1.3290	0.973
**ItV4**	3.595	1.3493	0.978

**Table 3:** Results of one-way ANOVA of H1–H4.

Dependent Variable	df	F-Value	Central Route	N	M	S.D	Scheffe’s Test	Result
**Perceived informativeness (H1)**	3	55.559	HH (S1)	68	4.294	0.888	>HL ***, LH, LL ***	Supported
			HL (S2)	54	2.389	1.238	>LL	
			LH (S3)	62	4.005	1.071	>HL ***, LL ***	
			LL (S4)	76	2.377	1.196	-	
**Perceived persuasiveness (H2)**	3	49.766	HH (S1)	68	4.229	0.865	>HL ***, LH, LL ***	
			HL (S2)	54	2.482	1.299	>LL	
			LH (S3)	62	3.723	1.038	>HL ***, LL ***	
			LL (S4)	76	2.295	1.111	-	
**Dependent variable**	df	F-Value	Peripheral Route	N	M	S.D	Scheffe’s Test	Result
**Perceived informativeness (H3)**	3	14.836	HH (S5)	74	3.756	0.849	>HL, LH, LL ***	Supported
			HL (S6)	68	3.711	0.873	>LL ***	
			LH (S7)	62	3.755	1.092	>HL, LL ***	
			LL (S8)	64	2.700	1.217	-	
**Perceived persuasiveness (H4)**	3	16.837	HH (S5)	74	4.026	0.801	>HL, LH, LL ***	
			HL (S6)	68	3.988	0.825	>LL ***	
			LH (S7)	62	4.000	0.957	>HL, LL ***	
			LL (S8)	64	2.987	1.116	-	

Notes: *** *p* < 0.001. Symbol (>) indicate that the mean score of the respective scenario is higher than the other stated scenario.

**Table 4:** Path analysis of H5–H7.

Hypotheses		Standard Coefficient (r^2^)	*t*-Value	Result
**H5**	Perceived Informativeness → Attitude towards Vaccine	0.553 ***	6.329	Supported
**H6**	Perceived Persuasiveness → Attitude towards Vaccine	0.430 ***	4.969	Supported
**H7**	Attitude towards Vaccine → Intention to obtain Vaccination	0.754 ***	30.894	Supported

Notes: *** *p* < 0.001; standardized root mean squared residual (SRMR) = 0.022 (model fit); normal fit index (NFI) = 0.908.

## Supplementary Materials

The following supporting information can be downloaded at: https://www.mdpi.com/article/10.3390/ijerph19042378/s1. Construct Definition and Description.
