# Supplementary material for: How Does Social Media Influence People to Get Vaccinated? The Elaboration Likelihood Model of a Person’s Attitude and Intention to Get COVID-19 Vaccines
Source: Int J Environ Res Public Health. 2022 Feb 18;19(4):2378. doi: 10.3390/ijerph19042378 (PMC8872449; doi:10.3390/ijerph19042378)
Supplement: Supplementary file 1 [file ijerph-19-02378-s001.zip › ijerph-1532604-supplementary.pdf]

Supplementary Material

Construct Definition and Description

Construct Name	Description	Source
Information Completeness	The extent to which the vaccine information provided is of sufficient depth and breadth. In this study, the information need to contain:<br>• Working mechanism of vaccine<br>• Information on vaccine effectiveness during the past<br>• Islamic legal perspective (Malaysian context)	(Elkalmi et al., 2021; Palm et al., 2021; Shulman & Bullock, 2020)
Information Accuracy	The degree to which vaccine information provided is correct, unambiguous, and accurate	(Elkalmi et al., 2021; Palm et al., 2021; Shulman & Bullock, 2020)
Experience Sharing	The post needs to contain users firsthand experience of getting vaccination. It also needs to include story telling technique in describing the process	(Botsis et al., 2020; Cornwall, 2020)
Social Pressure	The post needs to contain message on prosocial responsibility of an individual towards his or her immediate family (e.g., parents, spouses, children)	(Jordan et al., 2020)
Perceived Informativeness	The extent to which the respondents believes that the posts message includes informational contents about the vaccine	(Chang et al., 2020; Elhadi et al., 2021)
Perceived Persuasiveness	The extent to which the received information can persuade the respondent to believe on the vaccination	(Cesario et al., 2004; Petravić et al., 2021)
Attitude Towards Vaccination	A respondent "*learned predisposition*" to respond in a consistently favourable or unfavourable manner toward the COVID-19 vaccine in general"	(Cordina et al., 2021; Langford, 2020)
Intention to Vaccinate	Respondents think that they intend to get COVID-19 vaccination in the near future	(Dodd et al., 2021; Langford, 2020; Piltch-Loeb et al., 2021)

Details of Instrument and Sources

Construct Name	Items	Source
Perceived Informativeness	The twitter post content provides timely information about COVID-19 vaccines<br>The twitter post content supplies relevant information about COVID-19 vaccines<br>The twitter post content is a good source of information about COVID-19 vaccines<br>The twitter post content clearly describes about COVID-19 features<br>The twitter post content clearly informs me on how COVID-19 vaccines work<br>The twitter post content provides useful information data about COVID-19 vaccines	(Chang et al., 2020; Elhadi et al., 2021)
Perceived Persuasiveness	I consider the twitter post content to be convincing<br>I consider the twitter post content to be persuasive<br>I consider the twitter post content to be believable<br>I consider the twitter post content information to be trustworthy<br>I consider the twitter post content information is influential	(Cesario et al., 2004; Petravić et al., 2021)
Attitude Towards Vaccination	I felt that the twitter post content influence me to take COVID-19 vaccine<br>I value the twitter post content in informing me the importance of COVID-19 vaccine<br>I am satisfied with the twitter post content information on the importance of COVID-19 vaccine	(Cordina et al., 2021; Langford, 2020)

	The twitter post content helps me on making decision to take COVID-19 vaccine<br>The twitter post content positively teaches me on the important of COVID-19 vaccine<br>The twitter post content improves my trust on COVID-19 vaccine<br>The twitter post content enhance my information on COVID-19 vaccine efficacy	
Intention to Vaccinate	I would consider taking COVID-19 vaccine after reading the twitter post<br>I intend to take COVID-19 vaccine after reading the twitter post<br>I would probably get COVID-19 vaccine after reading the twitter post<br>I am very interested to be vaccinated by COVID-19 vaccine after reading the twitter post	(Dodd et al., 2021; Langford, 2020; Piltch-Loeb et al., 2021)

Scenario Design

Abbreviation: - 22 questions, 4 manipulation check question

1. IC = Information Completeness
2. IA = Information Accuracy
3. ES = Experience Sharing
4. SP = Social Pressure

No	Scenario Type	Description
1	Central (High IC & Hi IA)	High IC<br>• Contain information on past vaccine usage effectiveness (e.g., how vaccine prevent polio, measles).<br>• Contain information on how vaccine work<br>• Contain on information status in the perspective of Islam<br>High IA<br>The information provided is accurate and it is based on the information provided by Malaysia Ministry of Health (MoH) and can be accessed through the official website https://www.vaksincovid.gov.my/
2	Central (High IC & Low IA)	High IC – maintain all three information<br>Low IA – some of the statement if completely inaccurate but the statement is ensured to not mislead the respondent
3	Central (Low IC & High IA)	Low IC – some of the information is omitted (no info on past vaccine effectiveness and vaccine working mechanism)

		High IA – the retain information is accurate
4	Central (Low IC & Low IA)	Low IC – some of the information is omitted (no info on past vaccine effectiveness and vaccine information status in the perspective of Islam)<br><br>Low IA – some of the statement if completely inaccurate but the statement is ensured to not mislead the respondent
5	Peripheral (High ES & High SP)	High ES – Include firsthand experience with the COVID-19 vaccine. And include story telling technique in conveying the message.<br><br>High SP - contain message on prosocial responsibility of an individual towards his or her immediate family (e.g., parents, spouses, children)<br><br>Contain Verified profile, High RT, and Picture to get vaccinated
6	Peripheral (High ES & Low SP)	High ES – Include firsthand experience with the COVID-19 vaccine. And include story telling technique in conveying the message.<br><br>Low SP – does not contain message on prosocial responsibility<br><br>Contain common profile and no RT and No picture

7	Peripheral (Low ES & High SP)	Low ES – Does not contain firsthand experience with the COVID-19 vaccine. No story telling technique<br><br>High SP - contain message on prosocial responsibility of an individual towards his or her immediate family (e.g., parents, spouses, children)<br><br>Contain Verified profile, High RT, and Picture to get vaccinated
8	Peripheral (Low ES & Low SP)	Low ES – Does not contain firsthand experience with the COVID-19 vaccine. No story telling technique<br><br>Low SP – does not contain message on prosocial responsibility
